# Impact of IL-21-associated peripheral and brain crosstalk on the Alzheimer’s disease neuropathology

**DOI:** 10.1007/s00018-022-04347-6

**Published:** 2022-06-01

**Authors:** Sudhanshu Agrawal, Janet E. Baulch, Shreya Madan, Seher Salah, Samantha N. Cheeks, Robert P. Krattli, Veedamali S. Subramanian, Munjal M. Acharya, Anshu Agrawal

**Affiliations:** 1 Division of Basic and Clinical Immunology, Department of Medicine, University of California Irvine, Irvine, CA 92697 USA; 2 Department of Radiation Oncology, University of California Irvine, Irvine, CA 92697 USA; 3 Department of Anatomy and Neurobiology, University of California Irvine, Irvine, CA 92697 USA; 4 Division of Gastroenterology, Department of Medicine, University of California Irvine, Irvine, CA 92697 USA

**Keywords:** IL-21, Alzheimer’s disease, Neuroinflammation, Tfh, B1 cells

## Abstract

Alzheimer’s disease (AD) is associated with dysregulated immune and inflammatory responses. Emerging evidence indicates that peripheral immune activation is linked to neuroinflammation and AD pathogenesis. The present study focuses on determining the role of IL-21 in the pathogenesis of AD using human samples and the 5xFAD mice model. We find that the levels of IL-21 are increased in the periphery of both humans and mice in AD. In addition, the proportions of IL-21 target cells, Tfh and B plasma cells as well as activation of monocytes is increased in PBMCs from AD and mild cognitively impaired (MCI) subjects as compared to age-matched controls, indicating immune activation. In contrast, the percentage of B1 cells that control inflammation is decreased. These changes are due to IL-21 as the expression of IL-21 receptor (IL-21R) is higher on all these cells in AD. Furthermore, treatment with recombinant IL-21 in AD mice also leads to similar alterations in Tfh, B, B1, and macrophages. The effect of IL-21 is not confined to the periphery since increased expression of IL-21R is also observed in both humans and mice hippocampus derived from the AD brains. In addition, mice injected with IL-21 display increased deposition of amyloid beta (Aβ) plaques in the brain which is reduced following anti-IL-21R antibody that blocks the IL-21 signaling. Moreover, activation of microglia was enhanced in IL-21-injected mice. In keeping with enhanced microglial activation, we also observed increased production of pro-inflammatory cytokines, IL-18 and IL-6 in IL-21-injected mice. The microglial activation and cytokines were both inhibited following IL-21R blockage. Altogether, IL-21 escalates AD pathology by enhancing peripheral and brain immune and inflammatory responses leading to increased Aβ plaque deposition.

**Graphical abstract:**

IL-21 impacts AD neuropathology by enhancing peripheral and neuronal immune activation, inflammation, and Aβ plaque deposition. Increased levels of IL-21 in the circulation of AD and MCI subjects enhances the proportions of Tfh and B plasma cells indicative of peripheral immune activation. On the other hand, the proportions of B1 cells that help reduce inflammation and clear Aβ are reduced. In addition to the periphery, IL-21 also acts on the brain via IL-21 receptor, IL-21R that displays increased expression in the hippocampi of AD and MCI subjects. IL-21 enhances the activation of microglia, induces the secretion of pro-inflammatory cytokines and deposition of Aβ plaques in the brain in AD.

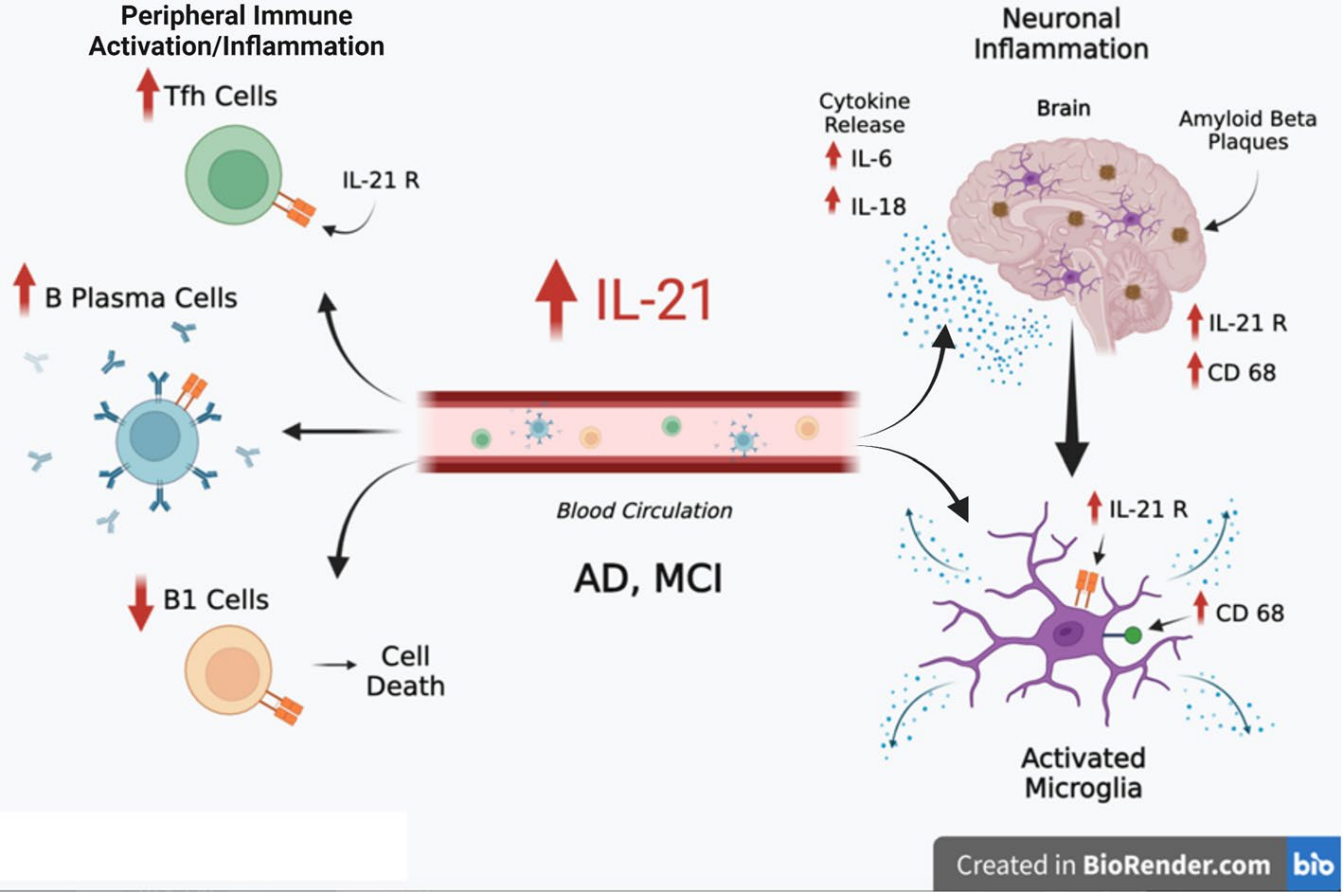

**Supplementary Information:**

The online version contains supplementary material available at 10.1007/s00018-022-04347-6.

## Introduction

Alzheimer’s disease (AD) is the most common form of dementia affecting the older population. The disease is devastating with no effective cure. Early diagnosis of the disease is also a major problem. The role of immune and inflammatory changes in AD pathogenesis [20, 39, 46, 47] is increasingly becoming apparent. Several recent genetic assays and reports underscore the importance of immune system genes in AD pathology. Despite the significant advances made in our understanding of immune system-mediated AD pathology, the changes in the peripheral immune cells and the concurrent effects on brain pathology is still an active area of investigation. It has become clear that the prominent role played by the immune system in AD is not limited to the brain. Copious evidence from clinical and experimental research suggests a significant role of the peripheral immune system and its responses in modulating AD pathogenesis. The central nervous system (CNS) has traditionally been thought of as an immune-privileged site. Yet, entry of peripheral immune cells into the brain via the blood-cerebrospinal fluid barrier (BCSFB) and brain lymphatics is thought to play an important role in immune surveillance and maintenance of a healthy CNS [15, 24, 34]. Estimates suggest that the cerebrospinal fluid (CSF) contains nearly 750,000 peripheral immune cells at any given time and over 90% of these surveilling cells are T cells, whereas the remaining 10% consist of B cells, and monocytes [31]. Thus, peripheral immune cells are constantly being exposed to and sampling CNS antigens and factors. Yet, their effects on brain cells and cognitive function are still understudied areas in AD. The cognate receptors for various inflammatory mediators on brain cells facilitates the transport of these mediators across the blood brain barrier. The prolonged immune activation and peripheral inflammation promotes cognitive decline and alterations in brain and peripheral immune cells in AD.

Most AD studies tend to focus on changes in functions of microglia in AD because they are the primary immune cells in the brain that play role in neuronal inflammation and disease pathology. In contrast, very few studies have examined the potential impact of adaptive immune cells in AD. This knowledge is essential since the adaptive immune system (T and B lymphocytes, etc.) and innate immunity (microglia, monocytes, etc.) rarely function independently of each other and both direct and indirect (cytokine- or antibody-mediated) crosstalk between these populations likely play an important role in AD. In support of this notion, recent studies have demonstrated profound effects of peripheral immune cells on pathogenesis in AD [9, 17, 38]. For example, higher levels of CD8+ T_EMRA_ were found in the blood of AD and mild cognitively impaired (MCI) patients and were associated with poor cognitive performance [17]. We also demonstrated that immune/inflammatory response to amyloid beta (Aβ) in the periphery is significantly higher in AD and MCI subjects as compared to healthy controls [2, 5]. We found increased levels of Immunoglobulin G (IgG) isotype and decreased levels of Immunoglobulin M (IgM) isotype of antibodies against Aβ in AD and MCI patients compared to controls. We also observed increase levels of the cytokine, Interleukin-21 (IL-21) in the serum of AD and MCI patients as compared to healthy age-matched controls. The AD mouse model 5xFAD too demonstrated significant increase in the inflammatory cytokine IL-21 in the spleen.

IL-21 is a highly inflammatory cytokine that belongs to the common gamma chain family of cytokines [32]. IL-21 is produced primarily by T-helper-17 (Th17) and T follicular helper (Tfh) cells. It acts on B lymphocytes to induce class switching from IgM to IgG and enhances antibody secretion [37]. Furthermore, IL-21 acts in an autocrine manner to enhance the generation of IL-17 producing inflammatory Th17 cells that have been demonstrated to play a role in a range of autoimmune/inflammatory disorders including multiple sclerosis, inflammatory bowel disease and psoriasis. The autocrine action of IL-21 also expands Tfh cells and dysregulated Tfh responses are implicated in allergic reactions, systemic autoimmune diseases, and chronic inflammation [22, 29]. Though we have observed an increase in IL-21 levels in AD both in humans and mice, the impact of this increase on AD pathogenesis has not been investigated. In the present study, using human patient samples and 5xFAD mouse model, we examined the crosstalk between the peripheral immune system, specifically IL-21 and the brain.

## Materials and methods

### Serum and PBMC samples from patients and controls

De-identified serum, cryopreserved peripheral blood mononuclear cells (PBMC) and brain (hippocampi) samples from AD (Alzheimer’s disease) and MCI (mild cognitive impaired) patients as well as age- and sex-matched healthy controls (HC) were obtained from the Alzheimer’s disease research center (ADRC) core at University of California Irvine (UCI). ADRC has an approved Institutional review board (IRB) protocol for sample collection. Participants enrolled in the UCI ADRC cohorts, undergo comprehensive at least annual evaluations that include a neurological and physical examination, neuropsychological assessment, brain imaging, lumbar puncture, blood and diagnostic tests, and an interview with a study partner. Table 1 provides the description of the samples.

**Table 1:** Description of AD, MCI cohort

	AD	MCI	HC
Number of subjects	34	34	34
Age range (years)	65–90	68–90	70–90
Age mean (years)	78.3	78.95	78.8
Gender			
Male	17	17	17
Female	17	17	17
MMSE scores	6–28	21–30	27–30
Mean ± SD MMSE	17 ± 4.78	27.3 ± 2.8	29.1 ± 1.01

*MMSE* the mini-mental state examination

### Mice

All animal experimentation procedures were performed in accordance with the guidelines provided by National Institutes of Health (NIH) and approved by the University of California Irvine Institutional Animal Care and Use Committee. Animals were maintained in standard housing conditions (20 °C ± 1 °C; 70% ± 10% humidity; 12 h:12 h light and dark cycle) and provided ad libitum access to standard rodent chow (Envigo Teklad 2020x) and water. We utilized the well characterized 5xFAD mouse model of AD that overexpresses mutant human APP (695) with the Swedish (K670N, M671L), Florida (I716V), and London (V717I) Familial Alzheimer’s Disease (FAD) mutations and human PS1 harboring two FAD mutations, M146L and L286V (MMRRC strain: 03448) for our studies [27]. These transgenic mice represent an accelerated AD model, developing AD pathologies by 2 months of age and reductions in synaptophysin, neuron loss and memory impairments that become evident by 5–6 months of age. We have used this AD model in our previous publications [3, 5, 40]. Early (1–3 months) male and female 5xFAD mice and their littermate controls were used for the experiments. In both cases the animals were stratified by age to maintain equivalent age distributions between experimental groups. Power analysis was performed to determine the number of animals needed for the experiments [13].

### IL-21 plasma assay

Plasma samples from AD, MCI and healthy controls were assayed for IL-21 by specific ELISA following the manufacturer’s instructions (RnD Systems, Minneapolis, MN).

### Flow cytometry of human PBMC

Cryopreserved PBMCs were thawed and left for 1 h at 37 °C in the CO_2_ incubator. Subsequently the cells were collected and stained with BD Horizon™ Fixable Viability Stain 510 to identify live cells, as per manufacture protocol. The cells were then washed, and surface stained for IL-21R PE expression on, (i) CD4, CD8 and Tfh (CD4/CD45RA -/CXCR5^+^/PD1^+^); (ii) B cell CD20, B1(CD20^+^CD27^+^CD43^+^), Plasma blast (CD20^+^CD38^+^) and iii) monocytes CD14^+^HLADR^+^.

After staining, the cells were washed and fixed using 2% PFA. Acquisition was done on BD FACSCelesta (Becton-Dickenson, San Jose, CA). Forward and side scatters and singlets were used to gate and exclude cellular debris. The flow cytometry results were analyzed using FlowJo™ v10.8 Software (BD Life Sciences, Ashland, OR). The details of the antibodies used are as follows: Fixable Viability stain510, CD4 FITC (clone: RPA-T4) from BD Bioscience (San Jose, CA), IL-21R PE (clone: 2G1-K12), CD8 PerCP (clone: SK1), PD1 APC (clone: EH12.2H7), CXCR5 BV421 (clone: J252D4), CD45RA BV605 (clone: HI100), CD38 BV421 (clone: HB-7), CD16 AF700 (clone: B73.1), CD14 BV650 (clone: M5E2), HLADR BV605 (clone: L243), CD20 PerCP (clone: 2H7), CD27 FITC (clone: M-T271), CD43 APC (clone: CD43-10G7) from BioLegend (San Diego, CA).

### IL-21 and anti-IL-21R injections in mice

3-month-old 5xFAD mice injected intravenously with recombinant mouse IL-21 (50 µg/kg) (BioLegend, San Diego, CA), twice weekly for a total of 5 injections to determine whether the IL-21 treatment would accelerate plaque deposition. Another group was injected with anti-IL-21R antibody (2.5 mg/kg) (Bio Xcell, Lebanon, NH) to block the action of IL-21 (IL-21R blocker) twice weekly for a total of 4 injections. 48 h after the last injection, mice were killed, and brains and spleens were collected for further analysis.

### Thioflavin S staining

Sections were rehydrated in an ethanol series (100%, 95%, 70%, 50%) and then incubated in a 0.5% thioflavin-S solution in 50% ethanol for 10 min. Tissues were rinsed twice in 50% ethanol and then rinsed twice in PBS. Sections were mounted and sealed with slow fade/gold antifade mounting medium (Life Technologies, Carlsbad, CA).

### Immunohistochemistry of mice brains

At 48 h post-IL-21 and anti-IL-21R antibody injections, mice were deeply anesthetized using isoflurane and euthanized via intracardiac perfusion using 4% paraformaldehyde (Sigma-Aldrich, St Louis, MO) in 100 mM phosphate buffered saline (PBS; pH 7.4, Thermo Fisher Scientific, Waltham, MA) [1]. Brains were cryoprotected (10–30% sucrose gradient over 2–3 days) and sectioned coronally into 30 µm using a cryostat (Microm HM525, Germany). For each endpoint, 4 representative coronal brain sections of the amygdala and medial prefrontal cortex (mPFC) regions from each of the 4 animals per experimental group were selected at approximately 15 section intervals and stored in PBS. For the immunofluorescence labeling of microglial activation marker CD68, rat anti-mouse CD68 (1:500; AbD Serotec, Hercules, CA) primary antibody was used with Alexa Fluor 594 secondary antibody (1:500). Similarly, for the IL-21R staining with microglia (IBA-1), we use IL21-R (BioXcell, Lebanon, NH) and rabbit anti-IBA-1 (Wako Chemicals, Richmond, VA) antibodies. The secondary antibodies included donkey anti-mouse or anti-rabbit Alexa Fluor 488 or 568. Tissues were then DAPI nuclear counterstained and sealed in slow fade/gold antifade mounting medium (Life Technologies, Carlsbad, CA).

### Confocal microscopy, image processing and 3D quantification

The stained coronal brain sections were scanned using a confocal microscope (Nikon Eclipse Ti C2) equipped with a 40 × PlanApo oil-immersion lens (1.3 NA, Nikon) and an NIS-Elements AR interface (v4.30, Nikon). 20–30 z stacks (1024-bit depth) at 0.5 µm from three different fields (318  × 318  × 24 µm) were imaged in each section in the areas of interest. The digitized z stacks were deconvoluted using the AutoQuant software (version X3.0.4, Media Cybernetics, Rockville, MD). An adaptive, 3D blinded method was used to create deconvoluted images for direct import into the Imaris module (version 9.0, Bitplane, Inc., Zurich, Switzerland). The 3D algorithm-based surface rendering and quantification of fluorescence intensity for each marker was carried out in Imaris at 100% rendering quality. For each marker (CD68, IBA-1, IL21-R, ThioS), each channel was analyzed separately where 3D surface rendering detects immunostained puncta or nuclear staining (DAPI) satisfying pre-defined criteria, for the puncta size (0.5–1 µm) verified visually for accuracy. Using deconvoluted confocal z stack volume from the control group (WT) as a baseline for the minimum thresholding, a channel mean intensity filter was applied and used for all the experimental groups for each batch of molecular markers. The pre-set parameters were kept constant throughout the subsequent analysis of immunoreactivity for each antigen. To maintain uniformity among the varying number of puncta for each individual time point and/or antigen analyzed, the number of puncta per 318 × 318 × 24 µm was normalized to WT control and data was expressed as mean immunoreactivity (percentage) relative to WT controls.

### Mouse brain cytokine assay

We have established an assay to determine levels of inflammatory mediators in the brain [26]. Freshly dissected hemi-brains (6 brains/group) were flash-frozen in liquid nitrogen. Subsequently, they were pulverized on dry ice and solubilized in PBS containing protease inhibitor cocktail. After centrifugation, the supernatants were collected and assayed for cytokines using a magnetic bead-based customized kit (ThermoFisher Scientific, Waltham, MA). The levels of the mediators (IL-1β, IL-17, IL-21, IL-1α, IL-6, CCL-5, TNF-α, CCL-2, IL-18) were determined and normalized to the amount of the protein in the lysate.

### Human and mouse brain qPCR for IL-21R

For human studies, RNA was extracted from the hippocampi of AD, MCI and HC. For mice studies, RNA was extracted from the brains of AD and WT untreated, and IL-21 treated mice using an RNA easy kit (Zymo Research, Irvine, CA). After conversion to cDNA (ThermoFisher Scientific, Waltham, MA), qPCR was performed using gene-specific primers for human (Forward 5′-CCCGACCTCGTCTGCTACA-3′; reverse 5′-TGGTCTTGCCAGGTAAGGGT-3′) and mouse (forward 5′-GGCTGCCTTACTCCTGCTG-3′; reverse 5′-TCATCTTGCCAGGTGAGACTG-3′) IL-21R. Beta-actin (human: forward 5′-CATCCTGCGTCTGGACCT; reverse 5′-TAATGTCACGCACGATTTCC-3′ and mice: forward 5′-ATCCTCTTCCTCCCTGGA-3′ and reverse 5′-TTCATGGATGCCACAGGA-3′ expression was used for normalization of qPCR data.

### Statistical analysis

GraphPad Prism software was used for statistical analysis. Unpaired Student’s *t* test was used for measuring significance within groups. For comparisons between three or more groups, one-way ANOVA followed by Tukey’s test was used. Values of *p* < 0.05 were considered significant. All tests were two tailed with 95% confidence interval.

## Results

### Comparison of the IL-21 plasma levels and percentages of B and T cells and activation of monocytes in AD, MCI and HC

**Fig. 1:**
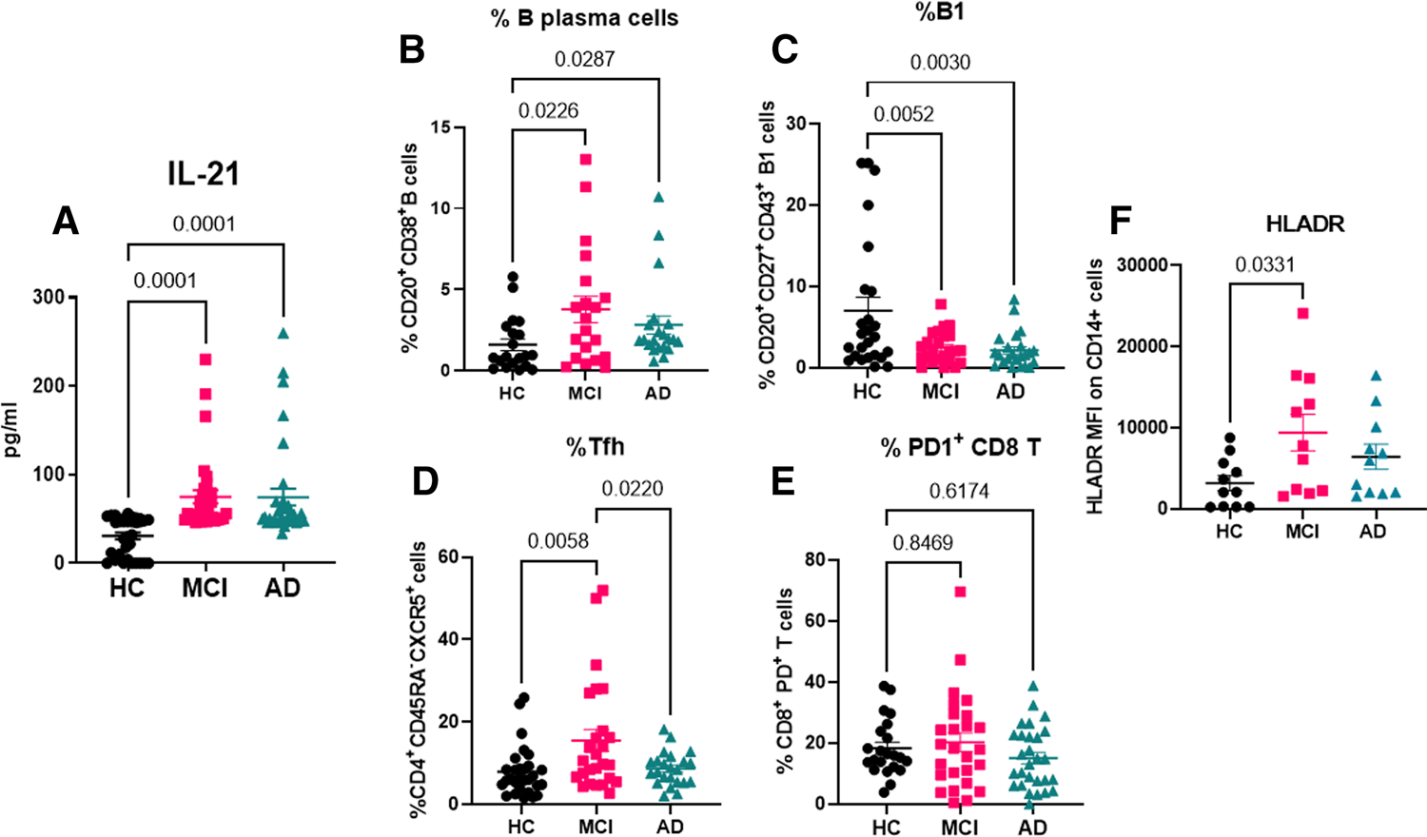
Comparison of the IL-21 plasma levels and percentages of B and T cells and activation of monocytes in AD, MCI and healthy controls. Plasma samples from AD, MCI and age-matched healthy controls (HC) were analyzed for IL-21 using specific ELISA. *N* = 34; *M* = 17; *F* = 17. **A** Dot plot depict the level of IL-21 in the three groups. PBMCs from AD, MCI and HC were subjected to flow cytometry and live gated cells were analyzed for the following—**B** B plasma cells (CD20^+^CD38^+^); **C** B1 cells (CD20^+^, CD27^+^, CD43^+^); **D** Tfh cells (CD4^+^CD45RA^−^CXCR5^+^); **E** exhausted CD8T cells (CD8^+^, PD1^+^); **F** activated monocytes (CD14^+^, HLADR^+^). *N* = 20; *M* = 10; *F* = 10. One way ANOVA followed by Tukey’s test was used for analysis

We have previously demonstrated that the level of cytokine IL-21 is increased in the plasma of AD and MCI patients as compared to controls [2]. A similar increase in IL-21 was observed in spleens of 5xFAD mice [5]. Here, we confirmed our findings in a larger cohort of patients. IL-21 levels were observed to be significantly increased in the plasma of MCI and AD patients as compared to healthy controls (Fig. 1A).

Next, we wanted to determine whether there were any changes in the proportions or phenotype of immune cells that are targeted by IL-21 in the PBMCs of AD, MCI and age-matched control patients. IL-21 is known to expand antibody producing B plasma cells. It also acts in an autocrine manner to enhance the expansion of T follicular helper (Tfh) cells. Flow cytometric analysis revealed that both B plasma cells (Fig. 1B) and Tfh cell (Fig. 1D) populations were expanded in MCI subjects as compared to healthy controls. Plasma B cells percentages were also increased in AD subjects as compared to controls (Fig. 1B). The total numbers of B and CD4 T cells were not significantly different between the three groups (data not shown). Gating strategy for flow cytometry is shown in Supplementary Fig. 1.

B cells are comprised of two major subsets, B1 and B2. B2 are the regular B cells that undergo class switch and produce IgG and other antibody isotypes. In contrast, B1 cells produce primarily IgM and play a major role in debris removal to prevent unwanted inflammation. We had previously observed a decrease in B1 cells in 5xFAD mice and this correlated with cognitive decline [5]. Here we determined whether a similar decrease in B1 cells was present in PBMCs of human AD and MCI subjects. We observed a significant decrease (*p* < 0.005) in B1 cell proportions in both AD and MCI subjects as compared to controls (Fig. 1C).

In infectious disease models, particularly chronic viral infections, IL-21 produced by CD4 T has emerged as an important modulator of CD8 T cell resident memory and exhausted cells development, homeostasis, and function. Since we were working with PBMCs, we compared the total number of CD8 T cells as well proportions of CD8 T cells expressing PD1 (exhausted) between the three groups. There was no significant difference either in total number of CD8 T cells (data not shown) or PD1^+^ CD8 T cells (Fig. 1E).

We had previously observed an increased activation in macrophages in 5xFAD mice [5]. For this reason, we next determined the changes in proportions and phenotype of monocytes as activation of these antigen presenting cells affects T and B cell responses in human patients. We did not observe significant differences in the numbers of monocytes in the PBMCs of AD, MCI and controls (data not shown). However, HLADR expression was significantly increased on the monocytes from MCI subjects as compared to control indicating an activated phenotype (Fig. 1F).

In all cell types, analysis was also performed based on sex, but the differences were not significant (data not shown).

Altogether, these data indicate that though the total number of B cells, CD4 T, CD8 T and monocytes were comparable in the circulation of AD, MCI and controls, specific subsets such as B plasma cells, B1 cells, Tfh cells and monocytes displayed significant changes in AD and MCI. It remains to be determined whether increased IL-21 contributed to these changes.

### IL-21R expression on cells in PBMCs of AD MCI and healthy controls

**Fig. 2:**
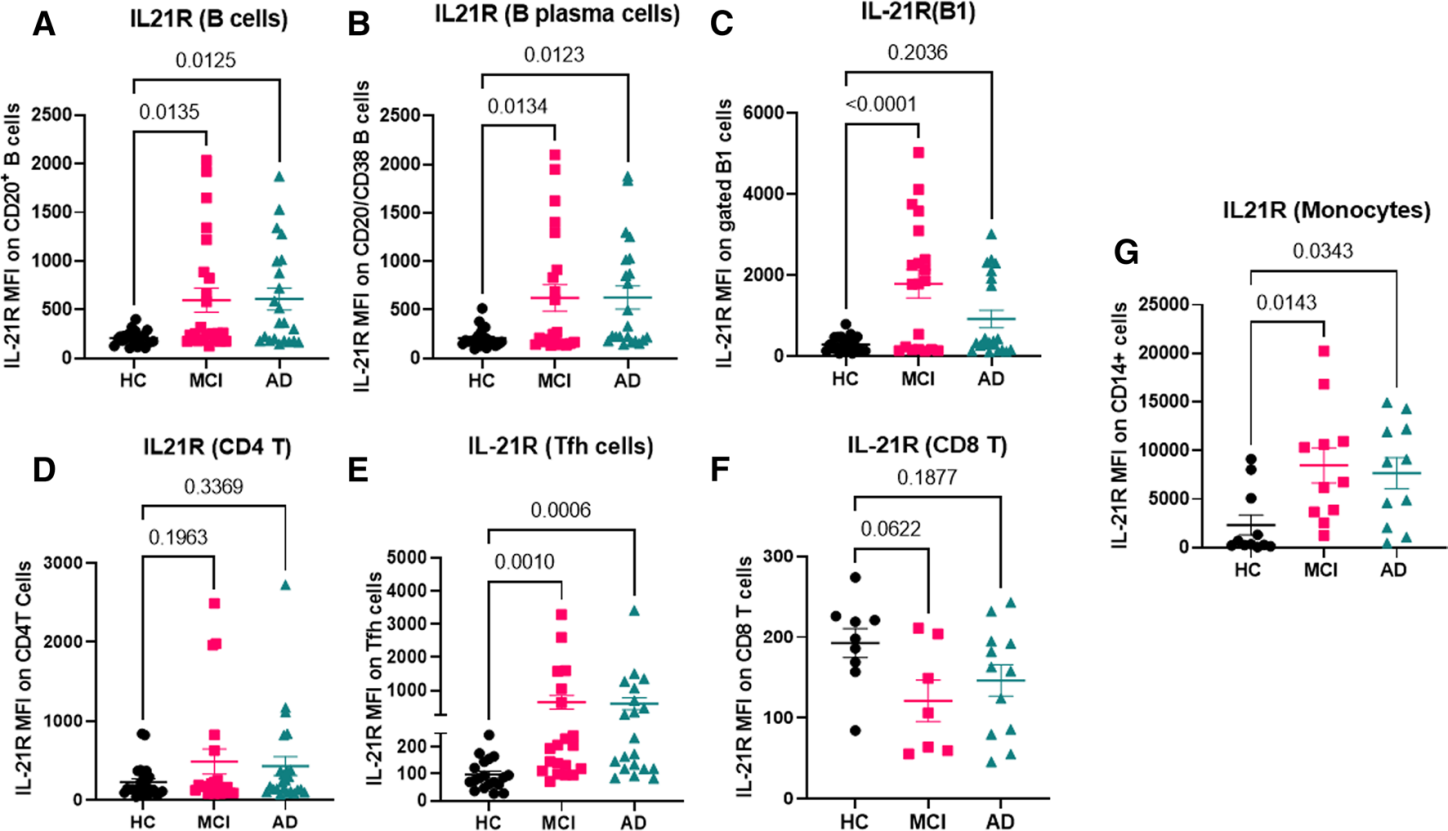
IL-21R expression on cells in PBMCs of AD MCI and healthy controls. IL-21R expression on immune cells was determined on PBMCs from AD, MCI and HC using flow cytometry. Dot plots depict the mean fluorescence intensity (MFI) of IL-21R on live gated cells. **A** Total B cells; **B** plasma B cells; **C** B1 cells; **D** total CD4 T cells; **E** Tfh cells; **F** CD8 T cells; **G** monocytes. *N* = 20; *M* = 10; *F* = 10. One way ANOVA followed by Tukey’s test was used for analysis

The biological functions of IL-21 are mediated by binding to its corresponding receptor, IL-21R that constitutes a complex with the common *γ*-chain. IL-21R is expressed by a wide range of immune cells, including T and B cells, and macrophages/microglia as well as non-immune cells, including astrocytes and neurons. We examined whether increased IL-21 levels in AD and MCI resulted in enhanced action of this cytokine on immune cells in the periphery by determining the expression of IL-21R on monocytes, T and B cells in the PBMCs of AD, MCI and HC via flow cytometry. The expression of IL-21R was significantly increased on B cells and B plasma cells in PBMC from MCI and AD patients as compared to HCs. IL-21 is known to act on B cells to induce antibody producing plasma cells so this data indicated that increased IL-21 in MCI and AD may be enhancing antibody production by B cells (Fig. 2A, B). This is in keeping with our previous studies where we observed increased levels of Aβ specific IgG antibodies in MCI and AD patients.

The effect of IL-21 on B1 cells is not known and we observed a decrease in B1 cells in MCI and AD, therefore we examined whether B1 cells express IL-21R and if this is altered in AD. Interestingly, IL-21R expression was also found to be significantly increased on B1 cells in both MCI and AD subjects as compared to HC (Fig. 2C). Similar increase in IL-21R expression was also observed on B1 cells in 5xFAD mice (Supplementary Fig. 2).

The expression of IL-21R on CD4 T cells was not significantly different between the three groups (Fig. 2D). However, when the CD4T cells were gated on T follicular helper (Tfh) cells, the expression of IL-21R was significantly increased in both MCI and AD PBMC as compared to HC (Fig. 2E). This is not surprising as Tfh cells not only are primary producers of IL-21 but are also the major target cells of IL-21. The expression of IL-21R on CD8 T cells was comparable between the three groups (Fig. 2F).

We also compared the expression of IL-21R on monocytes between MCI, AD and HC because they displayed an activated phenotype in MCI. Further, there are a few reports that indicate that IL-21 may enhance the phagocytic capacity of monocytes. IL-21R levels were significantly increased on monocytes from MCI and AD subjects as compared to HC (Fig. 2G).

Altogether, these data indicate that IL-21R levels are increased on Tfh, B plasma cells, B1 cells and monocytes in MCI and AD subjects as compared to HC. Thus, increased IL-21 in AD may be responsible for the changes in the proportions and phenotypes on these cells observed in Fig. 1.

### IL-21R expression in humans and mice AD brain

**Fig. 3:**
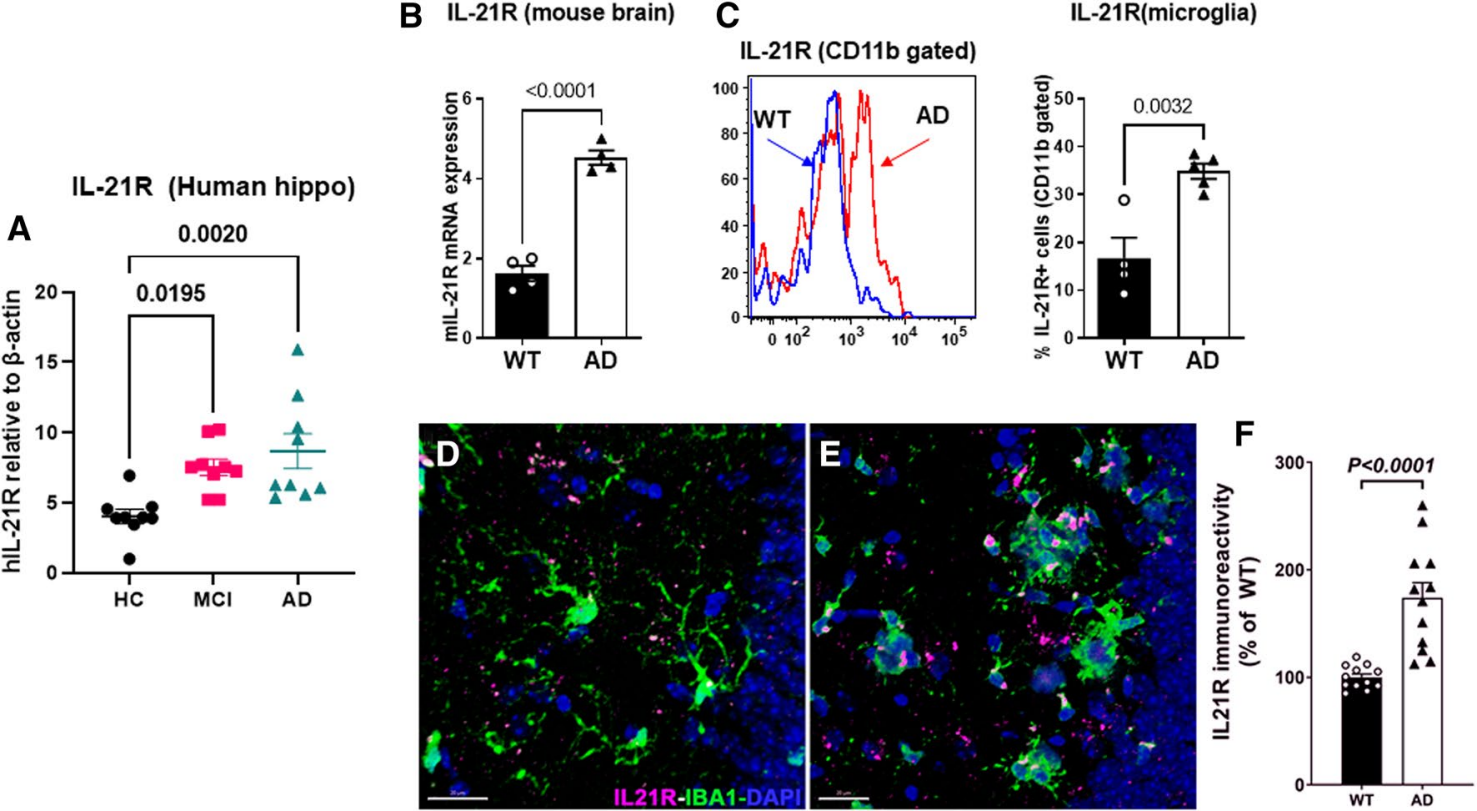
IL-21R expression in humans and mice AD brain. IL-21R expression was determined in the hippocampi of AD, MCI and HC using q-PCR. **A** Dot plot depicts the IL-21R gene expression; *N* = 9; **B** graph depicts the IL-21R expression in the hippocampi of 5xFAD mice and WT littermates using q-PCR; **C** IL-21R detection in the microglia of 5xFAD and WT mice by flow cytometry; IHC staining of IL-21R and microglia (IBA-1) was performed in the hippocampus of WT (**D**) and AD (**E**) mice; **F** plot depicts the volumetric quantification quantitation of IL-21R immunoreactivity

To determine whether peripherally produced IL-21 can affect brain cells, we compared the expression of IL-21R in the hippocampi of AD, MCI and age-matched HCs using qPCR. IL-21R expression was found to be significantly increased in both MCI and AD relative to controls (Fig. 3A).

Next, we determined whether an increase in IL-21R was also observed in 5xFAD mice. As is evident in Fig. 3B, IL-21R gene expression was significantly increased in the hippocampi of AD mice compared to WT littermate controls (*p* < 0.0001). Previous studies in mice have demonstrated the expression of the receptor for IL-21 (IL-21R) in the brain with maximal expression being observed on microglia, followed by neurons and astrocytes [11]. Flow cytometry data confirmed the increased expression of IL-21R on CD11b^+^ gated microglia in AD mice compared to WT controls (Fig. 3C). IHC staining and subsequent 3D algorithm-based volumetric analysis also showed that the proportion of CD11b^+^ microglia expressing IL-21R was significantly higher in AD mice (Fig. 3D).

### Administration of recombinant IL-21 enhances Aβ deposition in the brains of 5xFAD mice

**Fig. 4:**
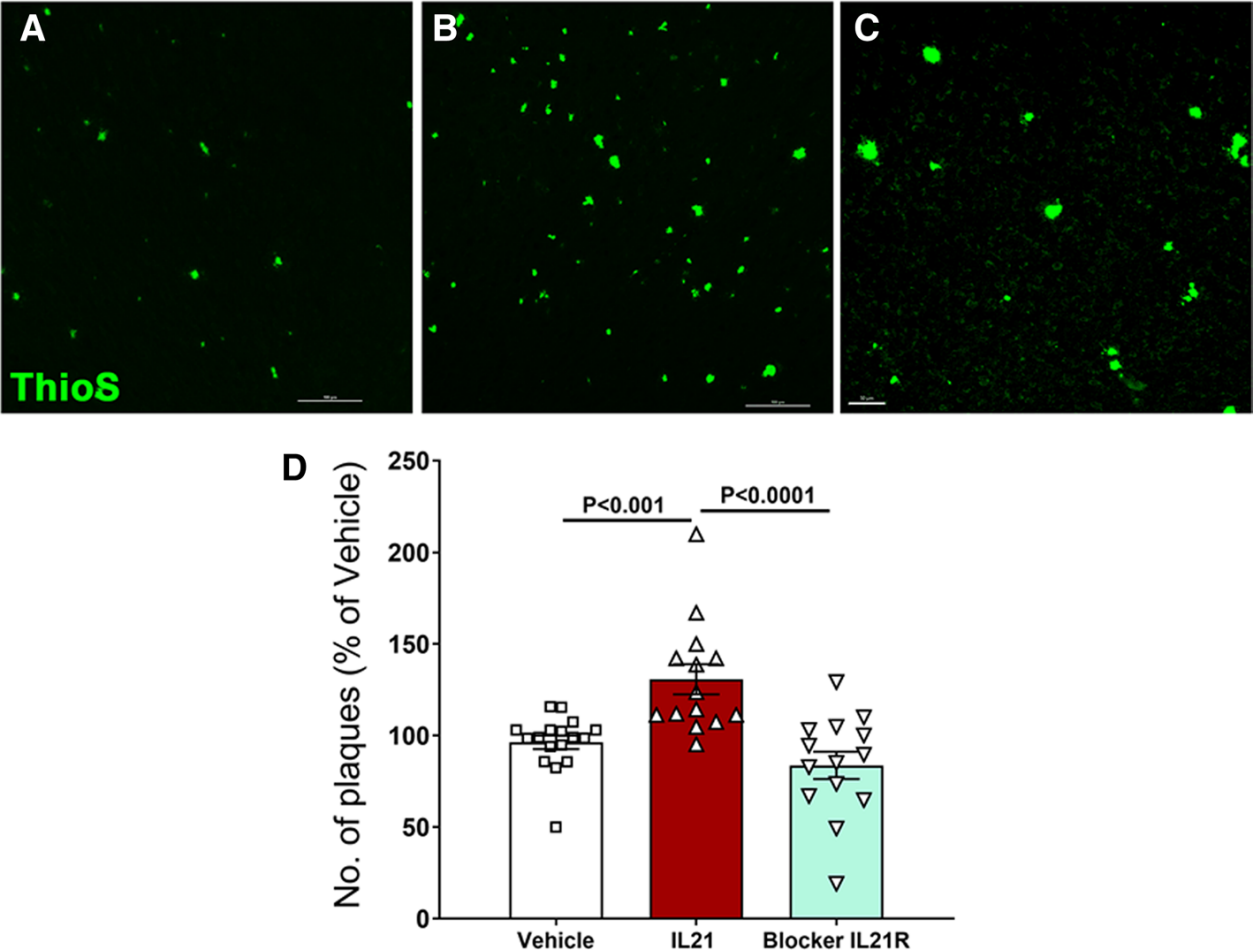
Administration of recombinant IL-21 enhances Aβ deposition in the brains of 5xFAD mice. Mice were given five injections of IL-21 or four injections of anti-IL-21R antibody (IL-21R blocker) and Aβ plaques were stained in the brain using thioflavin-S. Accumulation of plaques in—**A** vehicle-injected control; **B** IL-21 injected; **C** IL-21R blocker injected; **D** bar graph depicts the quantitation of the same in both males and females. *N* = 14

To evaluate the role of IL-21 in AD pathogenesis mice were injected with IL-21 and anti- IL-21R blocking antibody and effect on Aβ plaque deposition was determined. A significant increase in the number of plaques was observed in AD mice injected with IL-21 compared to vehicle (PBS)-injected mice (Fig. 4A–D) as well as IL-21R blocker. These data demonstrate that IL-21 plays a role in deposition of Aβ plaques and in the brain.

### Effect of IL-21 administration on neuroinflammation in 5xFAD mice

**Fig. 5:**
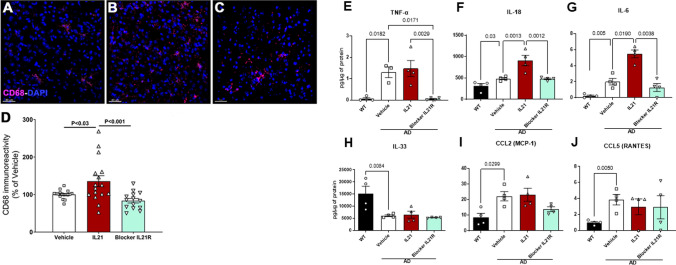
Effect of IL-21 administration on neuroinflammation in 5xFAD mice. Mice were given five injections of IL-21 or four injections of anti-IL-21R antibody (IL-21R blocker) and microglia were stained with CD68. CD68 staining in—**A** vehicle injected; **B** IL-21 injected; **C** IL-21R blocker injected; **D** bar graph depicts the quantitation of the same. The level of cytokines and chemokines were determined in the brain using multiplex. Bar graphs depict the pg/µg levels of—**E** TNF-α; **F** IL-18; **G** IL-6; **H** IL-33; **I** CCL-2; **J** CCL-5

We have previously reported increased CD68 (a lysosomal marker highly expressed on activated microglia) expression on the microglia in 5xFAD mice as compared to WT littermates [5]. To determine the effect of IL-21 on microglia in the brain we examined the expression of CD68, in the same mPFC area as the plaques in Fig. 4. Microglia in the IL-21-injected mice displayed significantly enhanced CD68 immunoreactivity compared to PBS-injected controls and IL-21R blocker (Fig. 5A–D). Increased activation of microglia by IL-21 will enhance neuroinflammation. We also examined the effect of IL-21 on neurodegeneration by staining for pre-synaptic protein, synaptophysin in the brains of AD- and IL-21-administered mice since this is decreased in AD as previously reported [3]. We found no significant difference in the level of synaptophysin between the three groups (Supplementary Fig. 3).

To further determine the effect of IL-21 on neuroinflammation, we measured the levels of cytokines and chemokines in the brains of 5xFAD mice injected with IL-21 and IL-21R blocker and vehicle as described previously [26]. As a reference control levels in WT littermates were also measured. The levels of pro-inflammatory mediators, TNF-α, IL-18 and IL-6 were significantly increased in vehicle-injected AD mice compared to WT littermates. IL-21 injection had no significant effect on TNF levels, but injection of IL-21R blocker resulted in a significant decrease bringing TNF-α to the levels observed in WT brains (Fig. 5E). IL-18 levels on the other hand were significantly increased in the IL-21-injected mice and blocked by IL-21R to the level of vehicle-treated AD mice (Fig. 5F). Like IL-18, IL-21 injection led to significantly increased IL-6 production that was blocked by IL-21R blocker (Fig. 5G). In contrast, while AD mice exhibited a significant decrease in IL-33 and increases in CCL-2 and CCL-5 relative to WTs, there was no significant effect of these mediators following IL-21 or IL-21R injection in AD mice (Fig. 5H–J).

In summary these data indicate that high levels of IL-21 can enhance neuroinflammation by activating microglia and increasing the production of TNF-α, IL-18 and IL-6.

### Effect of IL-21 administration on IL-21R expression and peripheral immune cells

**Fig. 6:**
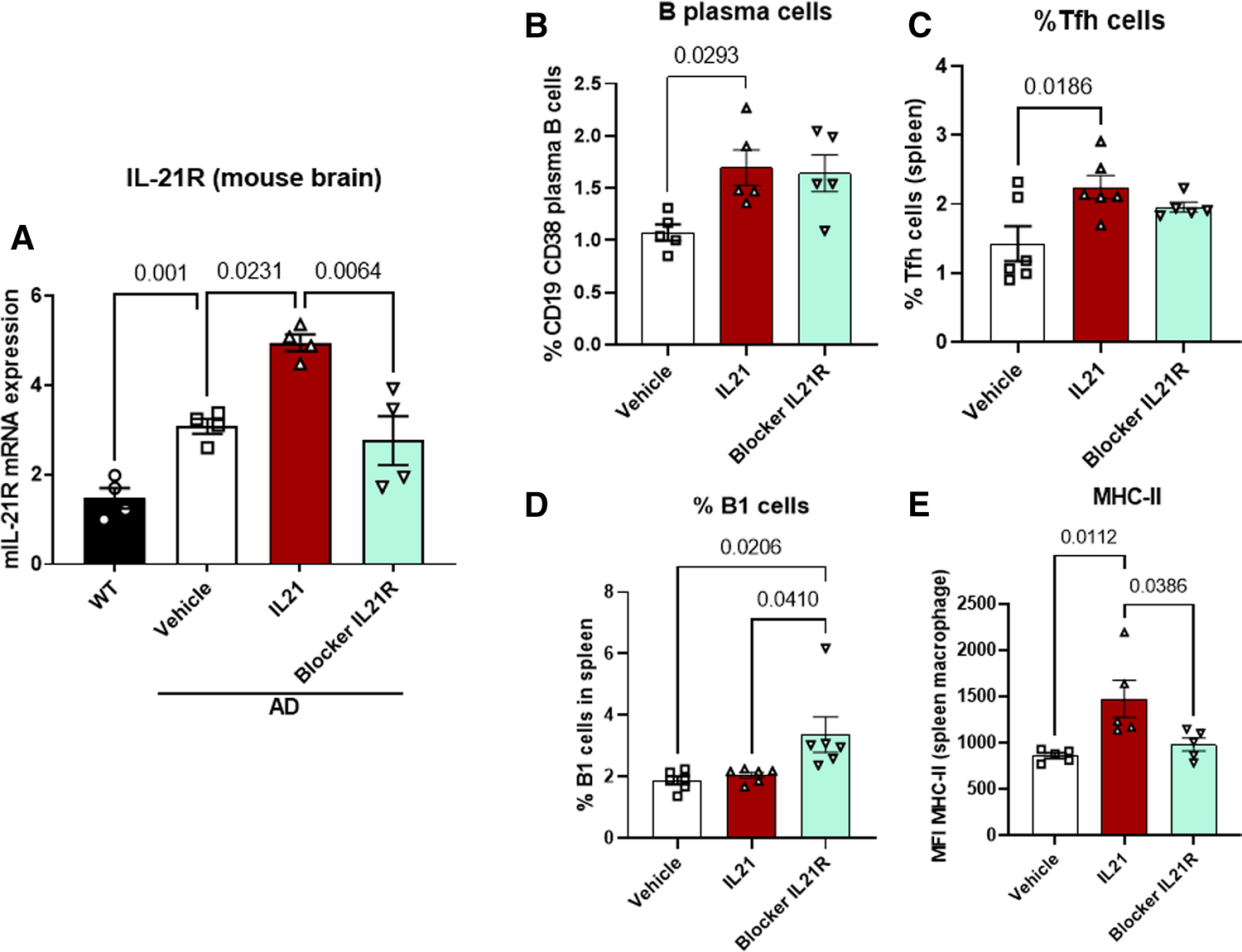
Effect of IL-21 administration on IL-21R expression and peripheral immune cells. Mice were given five injections of IL-21 or four injections of anti-IL-21R antibody (IL-21R blocker) and the following were determined. **A** IL-21R expression in the brain by qPCR; dot plot depict the percentages of cells in the spleen as determined by flow cytometry. **B** B plasma cells; **C** Tfh cells; **D** B1 cells; **E** MHC-II expression on macrophages

Next, we investigated whether changes in brain IL-21R expression and peripheral immune cells that were observed in human samples from AD and MCI patients were dependent on IL-21. To ascertain this, we determined the levels of IL-21R in mouse brain after injection with IL-21 and IL-21R blocking using q-PCR. IL-21 injection led to significant increase in IL-21R expression which was blocked by IL-21R antibody administration (Fig. 6A).

Investigation of peripheral immune cell changes in the mouse spleen after IL-21 injection revealed significant increases in B plasma cells and Tfh cells after IL-21 injection (Fig. 6B, C). The proportion of B1 cells was not affected by IL-21 injection however, blocking the action of IL-21 via IL-21R resulted in significant increase in B1 cells (Fig. 6D). Examination of macrophages revealed an increase in MHC-II expression following IL-21 injection indicating an activated phenotype similar to human monocytes in Fig. 1F. The activation was blocked by IL-21R (Fig. 6E).

These data indicate that IL-21 may be playing a role in the observed increase in brain IL-21R expression as well as alterations in B1, B plasma cells, Tfh cells and monocytes. Furthermore, increased IL-21 production during AD activates immune and inflammatory responses in the periphery as well as the brain.

## Discussion

The present project investigates the effect of IL-21 on AD pathogenesis. We have previously reported that IL-21 levels are increased in AD in both mice and humans. Here, we substantiated our previous findings in a larger cohort of human subjects. An increased frequency of IL-21 producing CD4^+^ T cells had been noted in AD [35] and the extent of brain atrophy was highly correlated with increased IL-21 producing CD4^+^ T cells [4]. Elevated levels of IL-17, IL-21 and IL-22 have also been described in AD patients [48]. Studies with other disease models likewise point to a role of IL-21 in modulating immune and inflammatoy responses in the CNS. For example, in IL-21R-deficient mice with experimental autoimmune uveitis (inflammatory eye disease), blocking IL-21 signaling was sufficient to attenuate CNS auto-inflammatory disease [44]. Increased IL-21 was also observed in the injured mouse brain after cerebral ischemia [11]. Further, IL-21-deficient mice exhibited smaller infarcts, better neurological function, and decreased lymphocyte infiltration into the brain. The CD4^+^ T cells infiltrating the brain were shown to be the main source of IL-21. Administration of IL-21R F_c_ fusion protein to these mice protected them from re-perfusion injury. IL-21 was identified as a mediator of brain injury in post-mortem human brain tissue too, where IL-21 was detected in perivascular CD4^+^ T cells in the area surrounding acute stroke lesions.

The major producers of IL-21 are Tfh and Th17 cells. Our results indicate that Tfh may be the source of increased IL-21 in AD and MCI human subjects as their numbers are increased in these groups. It is also possible that early IL-21 production is acting as a self-amplifying loop. Tfh cells are also one of target cells of IL-21, thus, IL-21 may be enhancing their induction in AD and MCI. This is supported by our data that show increased IL-21R expression on Tfh cells in AD and MCI. We also find enhanced differentiation of B cells into class-switched antibody secreting B plasma cells due to IL-21. This is in keeping with our previous studies where we observed increased Aβ specific IgG antibodies but decreased IgM isotype antibodies in AD and MCI subjects [2].

The decreased IgM response may be due to reduced number of B1 cells in AD and MCI subjects. B1 subset of B cells secretes IgM antibodies against autoreactive antigens such Aβ and helps in their removal without causing inflammation [19]. Though IgM antibodies have a low affinity against the antigen their avidity of binding to antigens is high because of their pentameric structure that aids in clearance of antigens. Further, IgM effectively binds the complement component C1q that also helps in clearance [10]. Natural autoantibodies against Aβ have also been demonstrated to play a protective role against AD [28, 30]. Autoantibodies against varying Aβ epitopes have been detected at higher levels in the blood of control subjects as compared to AD patients [30]. Thus, B1 may play an important role in restraining the initiation or progression of AD. We had previously reported a decrease in B1 cells in AD mice compared to WT littermates [5]. Our data also provide novel information about the effect of IL-21 on the B1 cells. There is virtually no information about the effect of IL-21 on B1 cells. There are some studies that show IL-21 causes the apoptosis of marginal zone B cells that are similar to B1 cells in many aspects [41]. We show that B1 cells not only express IL-21R but that blocking IL-21R restores the levels of B1 cells in AD mice. Thus, IL-21 may play a role in decreasing the number of B1 cells and hence IgM and reduced clearance of Aβ in AD.

Though the literature suggests a role of IL-21 in inflammatory disorders of CNS [11, 18, 33, 44], its effect in AD particularly on Aβ plaques has not been studied. Our data clearly indicates that IL-21 injection enhances Aβ plaque formation in AD mice. This effect is acute and rapid as the number of plaques are increased within just 17 days of five injections. Increased IL-21 in early stages may therefore, be enhancing Aβ deposition in the brain that leads to cognitive deficits.

Our results also indicate that IL-21 induces significant neuroinflammation since IL-21R expression is increased on the microglia displaying an activated phenotype. The action of IL-21 on microglia may be a protective mechanism to enhance clearance of Aβ since reports indicate that IL-21 enhances the phagocytic capacity of monocytes/macrophages [42, 43]. However, the downside is that the the microglia are activated and secrete pro-inflammatory cytokines. In keeping with this, we find increased secretion of pro-inflammatory mediators, IL-18 and IL-6 in the brains of IL-21-injected mice. IL-18 has important functions in the brain [6] and activated microglia are considered the major source of IL-18. IL-18 produced acts in an autocrine manner to induce the production of inflammatory cytokines, TNF-α and IL-1β from microglia [12]. Furthermore, IL-18 also induces the expression of FAS ligand (FAS-L) on microglia, oligodendrocytes, and astrocytes which enhances Fas-mediated neuronal apoptosis during inflammation [7]. Similar to IL-18, IL-21 injection enhances IL-6 production in the brains of AD mice. IL-6 is also produced by activated microglia and increased IL-6 levels have been observed in *post-mortem* human AD brains. Furthermore, IL-6 is reported to be a component of early-stage Aβ plaque formation [21] and is associated with synapse loss and learning deficits in AD mice [8]. Inhibition of pSTAT3 signaling, affecting IL-6 signaling, in the brains of AD mice was demonstrated to reduce memory impairments [25]. In contrast to IL-6 and IL-18, the production of TNF-α was not affected by IL-21, however, blocking of IL-21 signaling via IL-21R led to a significant reduction in TNF-α levels in the brains of AD mice. Interestingly, similar to IL-6 and IL-18, TNF-α is also a product of activated microglia and is known to enhance memory impairment in mouse models of AD [14, 23, 45]. Our data suggest that IL-21 signaling in microglia may be involved in TNF-α production from activated microglia. IL-33 was also one of the cytokines tested. It has been reported to be neuroprotective as it inhibits neuronal cell death, enhances cognitive function and phagocytic activity of microglia, and reduces Aβ levels [16]. In addition, IL-33 has also been shown to reduce neuroinflammation in AD [16, 36]. Administration of IL-21 had no change on the level of IL-33 highlighting the detrimental effect of IL-21 in AD.

In summary, we now show that IL-21 production is enhanced in the periphery of human AD subjects even at early stages (MCI) perhaps due to the activation of the immune system. The peripheral increase in IL-21 not only further activates the immune system causing increase in IL-21 producing Tfh cells along with increased antibody producing plasma cells but also enhances neuroinflammation and Aβ plaque deposition in the brain. Blocking of IL-21 signaling was able to alleviate the neuroinflammation and Aβ plaque formation suggesting a possible strategy for early intervention in AD.

### Supplementary Information

Below is the link to the electronic supplementary material.

Supplementary file1 (TIF 2420 kb)

Supplementary file2 (TIF 369 kb)

Supplementary file3 (TIF 677 kb)

## Data Availability

Data will be made on reasonable request.
