# Supplementary figures and images for: Impact of IL-21-associated peripheral and brain crosstalk on the Alzheimer’s disease neuropathology
Source: Cell Mol Life Sci. 2022 Jun 1;79(6):331. doi: 10.1007/s00018-022-04347-6 (PMC9160131; doi:10.1007/s00018-022-04347-6)

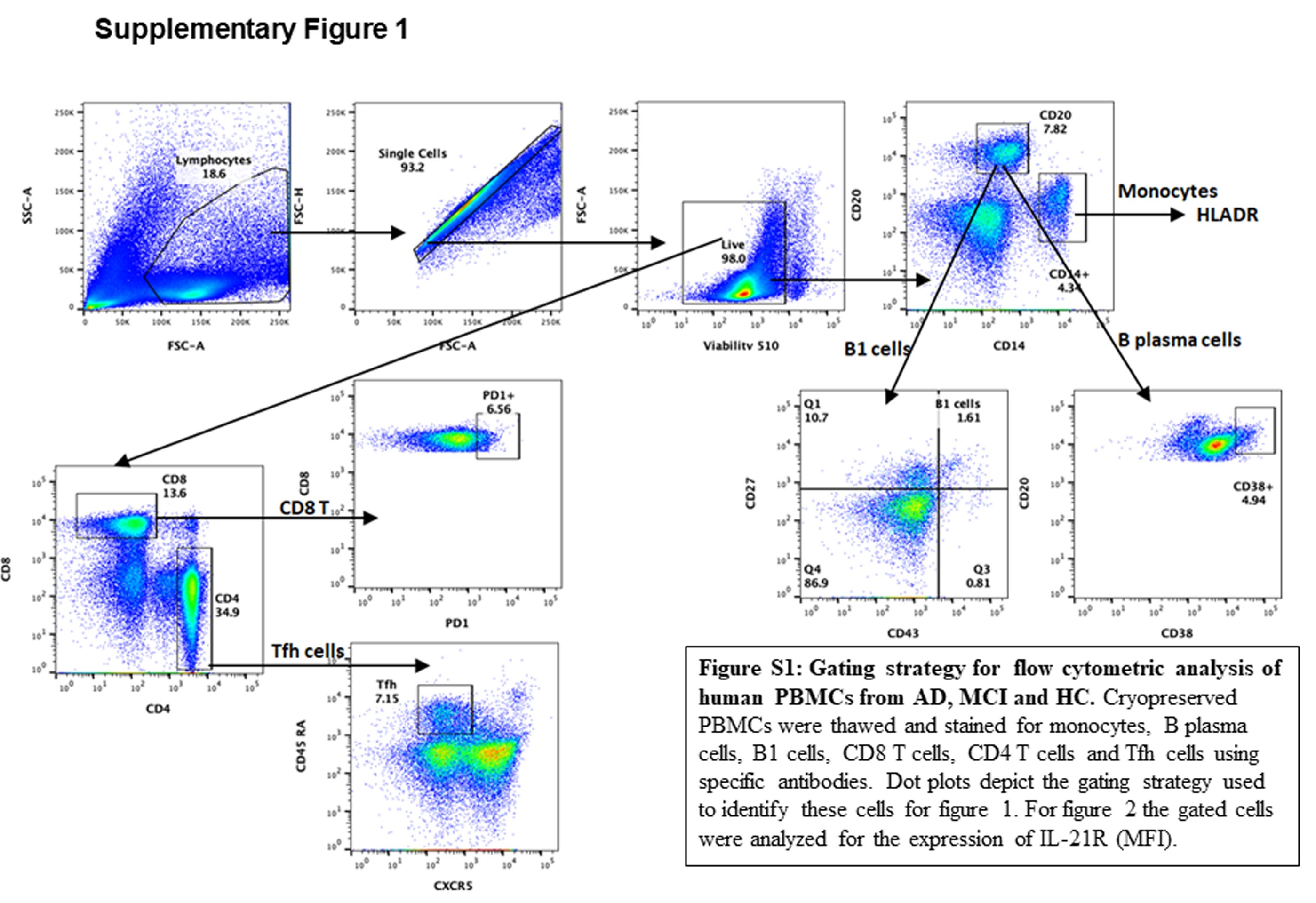

Supplement: Supplementary file 1 — Supplementary file1 (TIF 2420 kb) [file 18_2022_4347_MOESM1_ESM.tif]

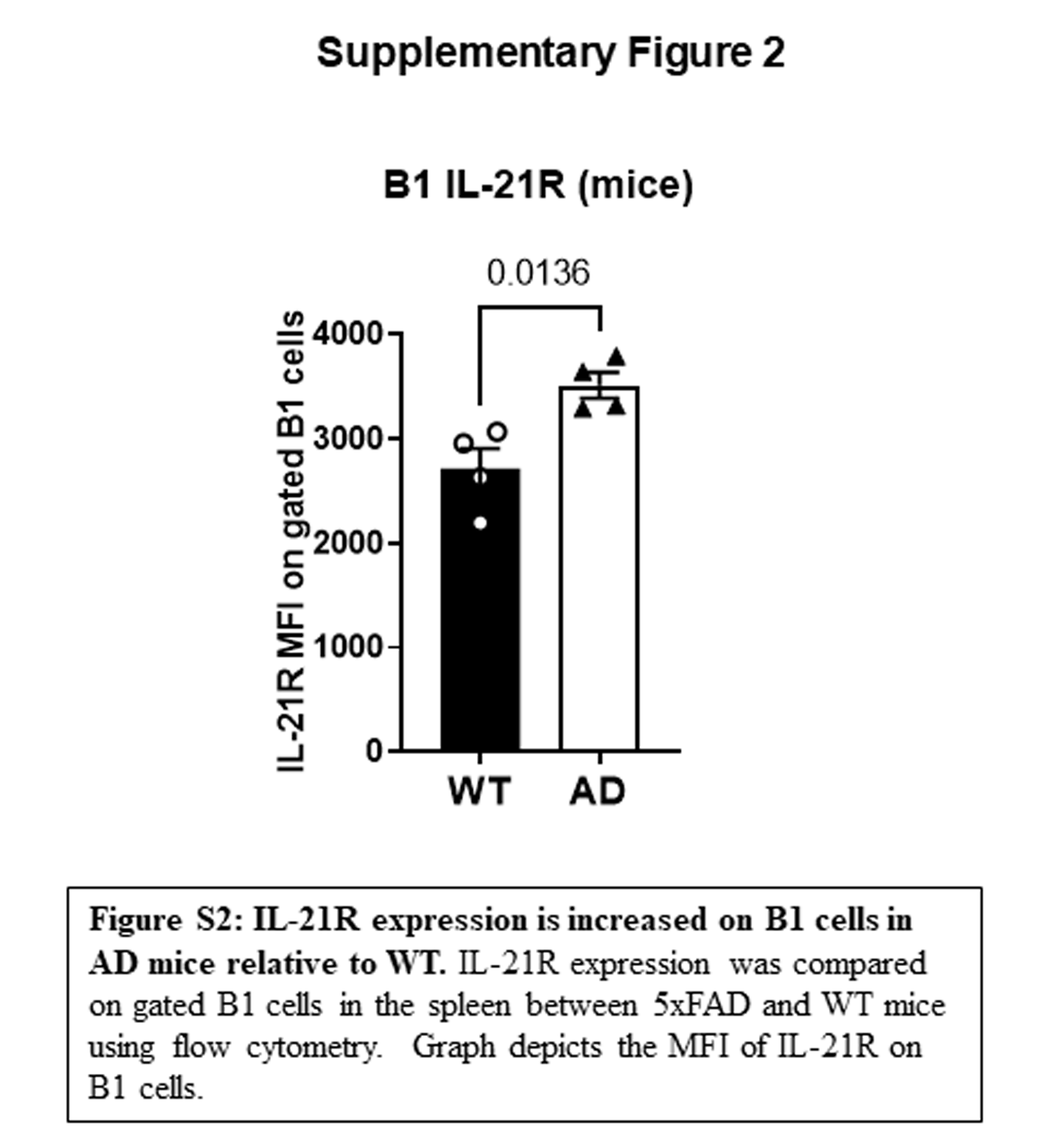

Supplement: Supplementary file 2 — Supplementary file2 (TIF 369 kb) [file 18_2022_4347_MOESM2_ESM.tif]

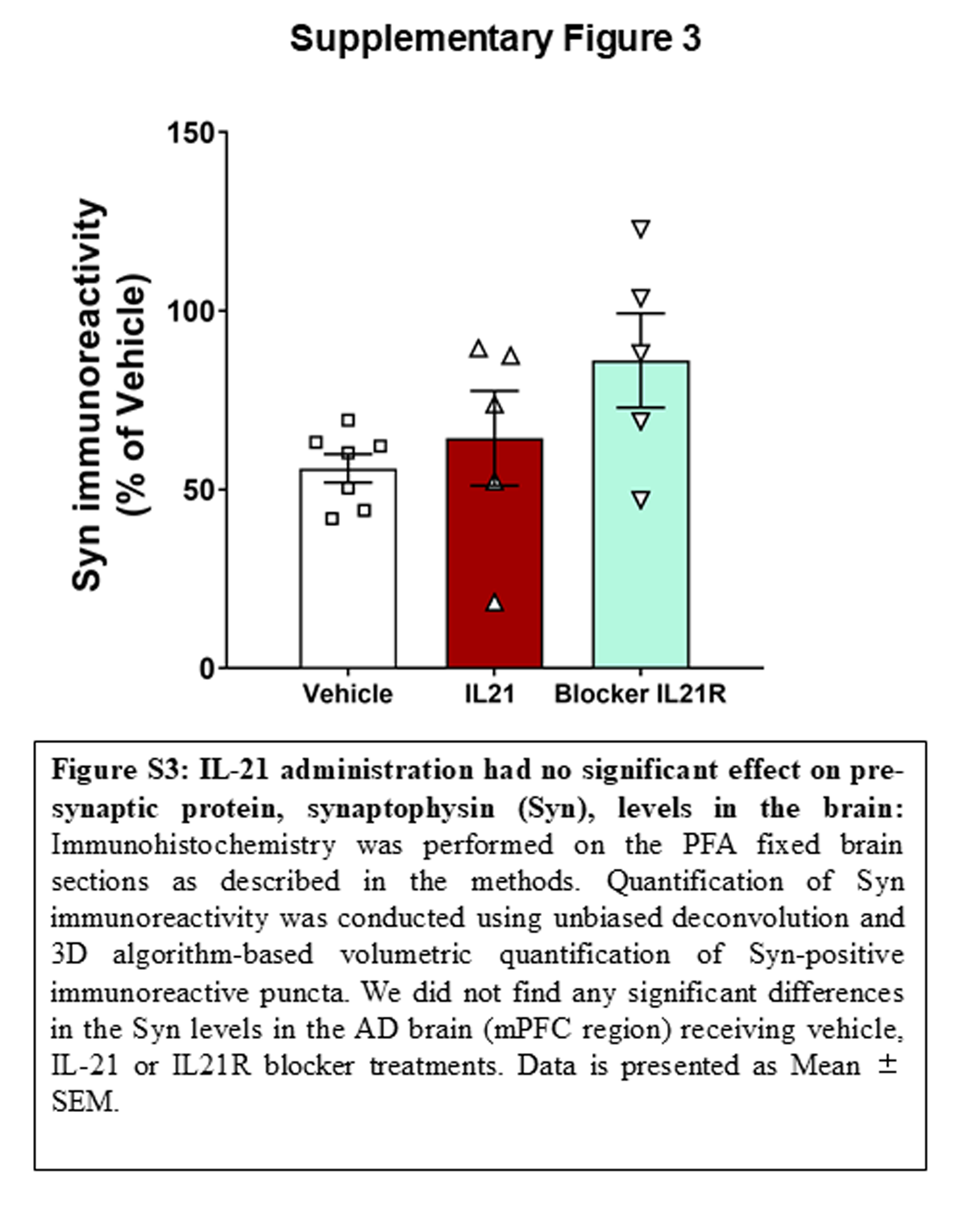

Supplement: Supplementary file 3 — Supplementary file3 (TIF 677 kb) [file 18_2022_4347_MOESM3_ESM.tif]
